# Comparative Studies on Different *Citrus* Cultivars: A Revaluation of Waste Mandarin Components

**DOI:** 10.3390/antiox9060517

**Published:** 2020-06-12

**Authors:** Giulia Costanzo, Maria Rosaria Iesce, Daniele Naviglio, Martina Ciaravolo, Ermenegilda Vitale, Carmen Arena

**Affiliations:** 1 Dipartimento di Biologia, Università degli Studi di Napoli Federico II, Via Cinthia, 80126 Napoli, Italy; giul.costanzo@studenti.unina.it (G.C.); ermenegilda.vitale@unina.it (E.V.); 2 Dipartimento di Scienze Chimiche, Università degli Studi di Napoli Federico II, Via Cinthia, 80126 Napoli, Italy; iesce@unina.it (M.R.I.); naviglio@unina.it (D.N.); martinaciaravolo@gmail.com (M.C.)

**Keywords:** ascorbic acid, antioxidant activity, chlorophyll and carotenoid content, phenolic compounds

## Abstract

Peel, pulp and seed extracts of three mandarin varieties, namely Phlegraean mandarin (*Citrus reticulata*), Kumquat (*Citrus japonica*), and Clementine (*Citrus clementina*) were compared and characterised in terms of photosynthetic pigment content, total polyphenols amount, antioxidant activity and vitamin C to assess the amount of functional compounds for each cultivar. The highest polyphenols content was found in the Phlegraean mandarin, especially in peel and seeds, whereas Kumquat exhibited the highest polyphenols amount in the pulp. The antioxidant activity was higher in the peel of Phlegraean mandarin and clementine compared to Kumquat, which showed the highest value in the pulp. The antioxidant activity peaked in the seeds of Phlegraean mandarin. The vitamin C in the Phlegraean mandarin was the highest in all parts of the fruit, especially in the seeds. Total chlorophyll content was comparable in the peel of different cultivars, in the pulp the highest amount was found in clementine, whereas kumquat seeds showed the greatest values. As regards total carotenoids, peel and pulp of clementine exhibited higher values than the other two cultivars, whereas the kumquat seeds were the richest in carotenoids. Among the analysed cultivars Phlegraean mandarin may be considered the most promising as a source of polyphenols and antioxidants, compared to the clementine and Kumquat, especially for the functional molecules found in the seeds. Moreover, regardless of cultivars this study also highlights important properties in the parts of the fruit generally considered wastes.

## 1. Introduction

In recent years, clinical trials and epidemiological studies have established an inverse correlation between the intake of fruits and vegetables and the occurrence of chronic diseases, the most prevalent causes of death in the world [1,2]. This protective effect has been ascribed to the antioxidant properties of different compounds, which coordinate and balance the body system to protect tissues and fluids from damage by reactive oxygen species (ROS) or free radicals [1,3,4].

Besides health and nutritional benefits, antioxidants have an important role for the food industry. These compounds prevent the propagation reaction of free radicals during the oxidative process preserving food quality and shelf life during handling and storage [5,6,7].

In general, citrus fruits are considered as one of the natural sources of antioxidants. In fact, they contain an appreciable amount of ascorbic acid, flavonoids, and phenolic compounds [8,9,10,11] and even some essential minerals important for human nutrition [12,13,14].

Mandarin is a product with many desirable characteristics for consumers who are health aware [15]. Continuous improvements in transportation logistics have allowed its widespread distribution to consumers throughout the world. These reasons have increased the world demand for mandarin cultivation so that its products are in continuous growth [16].

In food manufacturing, citrus is mainly used for producing fresh juice or citrus-based drinks, so a large amount of citrus wastes such as peels and seeds must be discarded. The global volume of citrus processed every year is about 31.2 million tons [17], 50%–60% of which represents waste called “pastazzo” [18,19]. The management of a such amount of wastes represents a critical issue for the citrus industry due to the high costs involved for its disposal [18,20].

This encourages the implementation of recycling policies to promote potential new and innovative uses of citrus by-products. Currently, several technological innovations have been developed to valorise citrus wastes in order to convert possible environmental risks into a valuable resource, thus reducing the environmental impact [19]. Citrus by-products find utilisation in biogas production [21], ruminant feeding [22], and essential oil extraction [23].

Moreover, a very interesting perspective would be also to utilise citrus by-products as a source of bioactive compounds for human diet [24,25]. In fact, recent studies suggest that citrus waste could be used as natural sources of antioxidants [26,27]. However, it is well known that the chemical composition of fruits may be subjected to variations according to climate, cultivation practices, soil type, cultivar, fruit maturity, and even between parts of the same fruit [1]. In addition to the expected changes of fruit quality, variations in antioxidant properties during ripening have also been described [28,29]. For a revaluation of citrus by-products, it would be appropriate to focus the attention on varieties with very different provenance and traits, in order to have an indication about the potential associated to the wastes of diverse cultivars.

Starting from these considerations, the aim of this study was to determine the amount of specific functional compounds such as chlorophyll and carotenoid, total polyphenols, vitamin C as well as the antioxidant capacity in the pulp and more specifically in peel and seeds of three different cultivars of mandarin, namely Phlegraean mandarin (*Citrus reticulata* Blanco), Kumquat (*Citrus japonica*), and Clementine (*Citrus × clementina*). These cultivars have been selected for specific characteristics. In detail, clementine was chosen for its large demand and commercial consumption worldwide due mainly to ease of peeling and seedlessness, kumquat is very appreciated in food preparation but has a niche consumption, while the Phlegraean mandarin is specifically diffused only in a peculiar volcanic area of Southern Italy (Campi Flegrei, Naples, Italy). The mandarin cultivar from the Phlegraean fields, planted for the first time in Naples in the 19th Century, is considered a traditional product from Campania region. The mandarin and the liqueur derived from it, namely “mandarinetto”, have been included by the Italian Ministry of Agriculture, Food and Forestry in the list of typical, traditional products of the Campania region (GU 168/2015). This citrus fruit variety has shown its maximum expression in the Phlegraean fields, a peculiar area surrounding the supervolcano, Vesuvius; the fertile soil, typical of a volcanic area as well as the proximity of the sea and the mild climate make the Phlegrean land unique and particularly favourable to the agriculture.

The outcomes of this study will be useful for a valorisation of mandarin waste products, encouraging their use and thus favouring the recycling practices and the bioeconomy strategies.

## 2. Materials and Methods

### 2.1. Plant Material and Sample Preparation

Three cultivars of mandarins (Phlegraean mandarin, *Citrus reticulata*; Kumquat, *Citrus japonica*; and Clementina, *Citrus clementina*) collected in the seasons 2019–2020 were used in this study. Fruits, used for the experiments, were homogeneously collected from five selected mandarin trees, for each cultivars. Phlegrean mandarin fruits were collected in the area of Bacoli, Phlegraean fields, (Naples, Southern Italy); mandarins from Clementine and Kumquat cultivars were sampled in private plantations at Sorrento peninsula (Naples, Southern Italy). Sampling was carried out during the harvest period typical for each cultivar, generally from November to January. The samples were placed in plastic bags and stored in dry ice, then they were transferred immediately to the laboratory and stored at −80 °C for subsequent analysis. Analyses were performed on each component of the fruit: peel, pulp and seeds for all the collected samples.

The fruits were washed with tap water, separated into the three components and homogenised, using mortar and pestle, by preventively powdering in liquid nitrogen. Powdered citrus tissues, obtained for each component of the fruit, were placed in test tubes and stored at −20 °C until analysis. At least three different trees for each mandarin cultivar were chosen to collect samples. For each mandarin cultivar a total of ten samples were analysed. Mandarins of two collections (seasons 2019 and 2020) for all cultivars were analysed in this study.

### 2.2. Photosynthetic Pigments Content Determination

Total chlorophylls and carotenoids contained in peel, pulp and seeds were determined according to Lichtenthaler (1987) [30]. Briefly, pigments were extracted from 0.25 g of powered sample in ice-cold 100% acetone and centrifuged (Labofuge GL, Heraeus Sepatech, Hanau, Germany) at 5000 rpm for 5 min. The absorbance was measured by spectrophotometer (Cary 100 UV-VIS, Agilent Technologies, Santa Clara, CA, USA) at wavelenghts of 470, 645 and 662 nm and pigment concentration expressed as mg g^−1^ fresh weight (FW).

### 2.3. Total Polyphenol Content

Total polyphenol content was measured according to the reported procedure [31]. Briefly, 0.25 g of powered sample was extracted with aqueous 80% methanol, at 4 °C (for 30 min) and then centrifuged at 11,000 rpm for 5 min. Extracts were combined with 1:1 (*v*/*v*) 10% Folin–Ciocalteu phenol reagent and water. After 3 min, 700 mM Na_2_CO_3_ solution was added to the resulting mixture in 5:1 (*v*/*v*). Samples were incubated for 2 h in darkness. Then, the absorbance at 765 nm was measured with a spectrophotometer (UV-VIS Cary 100, Agilent Technologies, Palo Alto, CA, USA). Gallic acid was used as a standard. Calibration curve was constructed analysing standard solutions in the interval of concentration 5–500 ppm. The total polyphenols concentration was calculated and expressed as gallic acid equivalents (mg GAE g^−1^ FW) from the calibration curve (*R*^2^ = 0.996) using gallic acid.

### 2.4. Determination of Antioxidant Capacity and Ascorbic Acid

The antioxidant activity of the cultivars was evaluated by the Ferric Reducing Antioxidant Power (FRAP) assay, according to the reported method [32]. Briefly, 0.25 g of powered sample was mixed with 60:40 (*v*/*v*) methanol/water solution and centrifuged at 14,000 rpm for 15 min (4 °C). FRAP reagents (300 mM Acetate Buffer pH 3.6; 10 mM tripyridyltriazine (TPTZ), 40 mM HCl and 12 mM FeCl_3_) were added to the extracts of each sample in 16.6:1.6:1.6 (*v*/*v*), respectively. After 1 h in darkness, the absorbance at 593 nm was measured with a spectrophotometer (UV-VIS Cary 100, Agilent Technologies, Palo Alto, CA, USA). Trolox (6-hydroxy-2,5,7,8-tetramethylchroman-2-carboxylic acid) was used as the standard and total antioxidant capacity was quantified and expressed as mmol Trolox equivalents (µmol Trolox Eq. mg^−1^ FW).

The ascorbic acid (AsA) content was determined using the Ascorbic Acid Assay Kit (MAK074, Sigma-Aldrich, St. Louis, MO, USA), following the reported procedure [33]. Briefly, 10 mg of sample was homogenized in 4 volumes of cold AsA buffer, and then centrifuged at 13,000 rpm for 10 min at 4 °C to remove insoluble material. The liquid fraction was mixed with AsA assay buffer to a final volume of 120 μL. The assay reaction was performed by adding the kit reagents to the samples.

In this assay, the AsA concentration was determined by a coupled enzyme reaction, which develops a colorimetric (570 nm) product, proportionate to the amount of ascorbic acid contained in the sample. The concentration of ascorbic acid in the samples was referred to a standard curve and expressed in mg L^−1^.

### 2.5. Statistical Analysis

Statistical analysis was performed using Sigma Plot 12.0 (Jandel Scientific, San Rafael, CA, USA). Statistically significant differences among varieties were checked by one-way ANOVA followed by Holm Sidak test for multiple comparison tests, based on a significance level of *p* < 0.05. The normal distribution of data was verified by Shapiro–Wilk and Kolmogorov–Smirnov tests. Spearman correlation coefficient was used to test associations between variables. All data were expressed as means ± standard error (SE) (*n* = 6).

## 3. Results

### 3.1. Total Polyphenol Content

**Figure 1:**
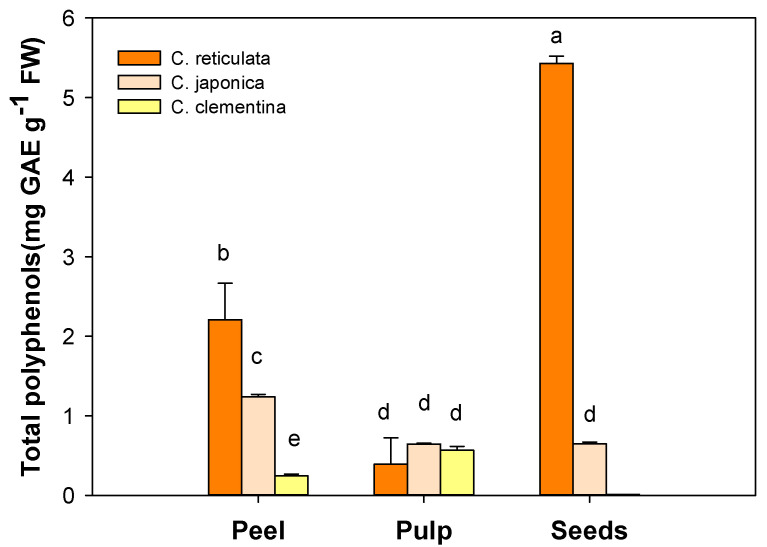
Total polyphenols in peel, pulp and seeds of the three different mandarin cultivars: *C. reticulata* (Phlegrean mandarin), *C. japonica* (Kumquat) and *C. clementina* (Clementine). Each bar represents the mean ± SE (*n* = 6). Different letters indicate statistically significant differences among mandarin varieties (*p* < 0.05). Results were analysed by one-way ANOVA followed by Holm–Sidak post hoc test for multiple comparisons. GAE: gallic acid equivalents; FW: fresh weight.

Total phenolic content of the peel, pulp and seeds extracts strongly varied among *Citrus* varieties and components of the fruit (Figure 1). Highest amounts (*p* < 0.05) of total polyphenols were found in *C. reticulata* peel (2.21 ± 0.19 mg GAE g^−1^ FW), followed by *C. japonica* (1.24 ± 0.013 mg GAE g^−1^ FW) and *C. clementina* (0.24 ± 0.011 mg GAE g^−1^ FW). No statistically significant difference was detected in the total polyphenol content for the pulp of all the examined Citrus varieties.

Among the fruit components, seeds of the *C. reticulata* cultivar exhibited the highest (*p* < 0.001) total polyphenol content (5.43 ± 0.04 mg GAE g^−1^ FW); this concentration was eight-fold higher than that measured in *C. japonica* seed extracts (0.65 ± 0.011 mg GAE g^−1^ FW).

### 3.2. Total Soluble Antioxidant Capacity

**Figure 2:**
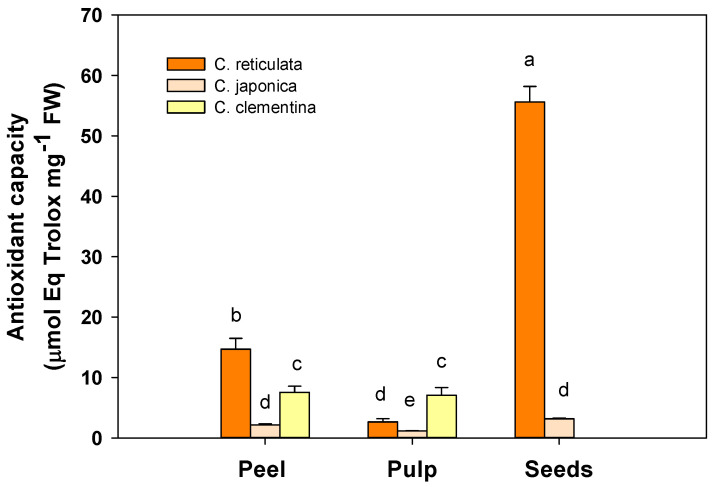
Total antioxidant capacity of peel, pulp and seeds in the three different mandarin cultivars: *C. reticulata* (Phlegrean mandarin), *C. japonica* (Kumquat) and *C. clementina* (Clementine). Each bar represents the mean ± SE (*n* = 6). Different letters indicate statistically significant differences among mandarin varieties (*p* < 0.05). Results were analysed by one-way ANOVA followed by Holm–Sidak post hoc test for multiple comparisons.

The total antioxidant capacity of the different cultivars is shown in Figure 2 for different parts of mandarin fruit: peel, seeds and pulp. The peel extracts of *C. reticulata* cultivar exhibited the highest (*p* < 0.01) total antioxidant capacity (14.69 ± 1.80 mmol eq Trolox mg^−1^ FW) compared to *C. clementina* (7.5 ± 1.0 mmol Trolox eq mg^−1^ FW) and *C. japonica*, which showed the lowest antioxidant capacity (2.1 ± 0.2 mmol Trolox eq mg^−1^ FW).

In the *C. clementina* pulp extracts, the total antioxidant capacity was found to be three-fold higher (7.1 ± 1.27 mmol Trolox eq mg^−1^ FW) (*p* < 0.01) than that measured in *C. reticulata* (2.6 ± 0.5 mmol Trolox eq mg^−1^ FW) and almost six-fold higher compared to *C. japonica* (1.2 ± 0.06 mmol Trolox eq mg^−1^ FW).

As observed for total polyphenols, among the different fruit parts, the seeds of *C. reticulata* showed the highest (*p* < 0.001) antioxidant capacity (55.6 ± 2.6 mmol Trolox eq mg^−1^ FW). This value was seventeen-fold more abundant compared to *C. japonica* (3.2 ± 0.15 mmol Trolox eq mg^−1^ FW).

### 3.3. Total Chlorophyll and Carotenoid Content

**Figure 3:**
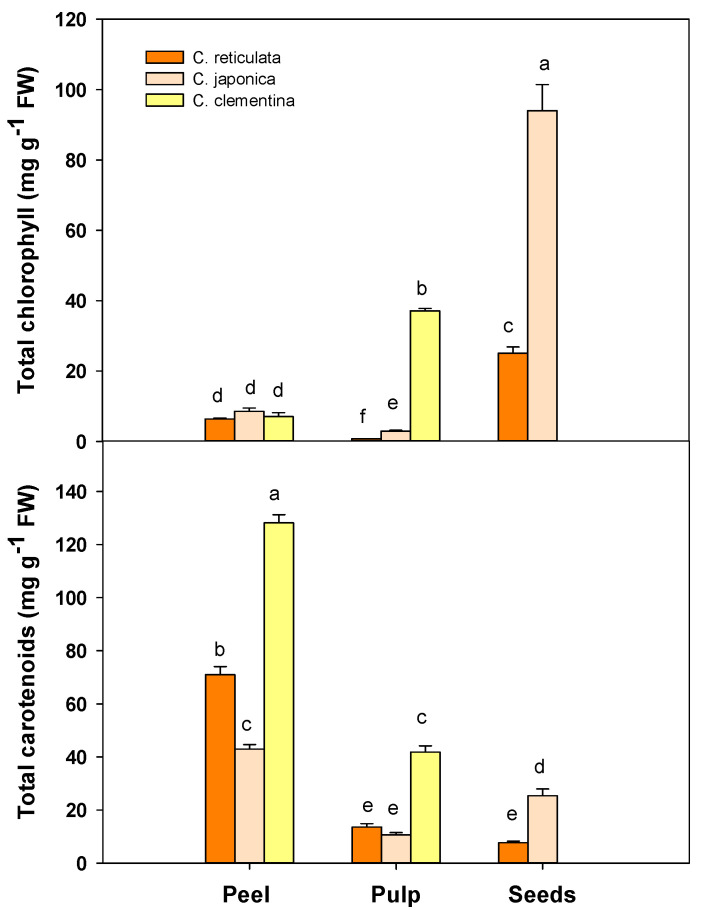
Total chlorophyll (a + b) and total carotenoid (x + c) content of peel, pulp and seeds in the three different mandarin cultivars: *C. reticulata* (Phlegrean mandarin), *C. japonica* (Kumquat) and *C. clementina* (Clementine). Each bar represents the mean ± SE (*n* = 6). Different letters indicate statistically significant differences among mandarin varieties (*p* < 0.05). Results were analysed by one-way ANOVA followed by Holm–Sidak post hoc test for multiple comparisons.

The concentration of pigments varied significantly among both cultivars and fruit components. Regarding peel, no difference in total chlorophyll content was detected among cultivars (Figure 3). Conversely, the total carotenoid content increased significantly (*p* < 0.01) in *C. clementina* peel (128.16 ± 3.03 mg g^−1^ FW) compared *C. reticulata* (70.99 ± 3.01 mg g^−1^ FW) and *C. japonica* (42.97 ± 1.72 mg g^−1^ FW) (Figure 3).

In the pulp of *C. clementina* cultivar the total chlorophyll content was very high (*p* < 0.01) (37.13 ± 0.67 mg g^−1^ FW) compared to values observed for *C. japonica* (2.93 ± 0.23 mg g^−1^ FW) and *C. reticulata* (0.73 ± 0.07 mg g^−1^ FW). The same trend was observed for carotenoid content in the pulp (Figure 3).

The total amount of chlorophyll increases significantly (*p* < 0.001) in *C. japonica* seeds (94.01 ± 7.92 mg g^−1^ FW), compared to *C. reticulata* (25.05 ± 1.78 mg g^−1^ FW). The highest concentration (*p* < 0.01) of carotenoids was detected in *C. japonica* seeds (Figure 3).

### 3.4. Ascorbic Acid

**Figure 4:**
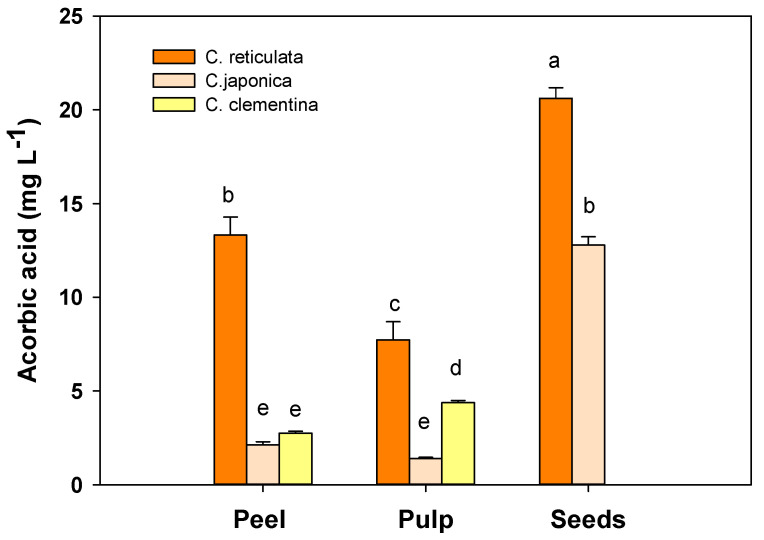
Total soluble antioxidant capacity expressed as ascorbic acid equivalents in peel, pulp and seeds in the three different tangerine cultivars: *C. reticulata* (Phlegrean mandarin), *C. japonica* (Kumquat) and *C. clementina* (Clementine). Each bar represents the mean ± SE (*n* = 6). Different letters indicate statistically significant differences among mandarin varieties (*p* < 0.05). Results were analysed by one-way ANOVA followed by Holm–Sidak post hoc test for multiple comparisons.

Ascorbic acid (AsA) content was very different among mandarin cultivars and fruit components. The highest values (*p* < 0.01) were found in peel, pulp and seed extracts of *C. reticulata* (Figure 4).

In particular, in the peel of *C. reticulata* the ascorbic acid concentration was higher (*p* < 0.01) (13.32 ± 0.96 mg L^−1^) compared to that measured in *C. clementina* (2.75 ± 0.10 mg L^−1^) and *C. japonica* (2.13 ± 0.16 mg L^−1^). In pulp the trend was the same with the highest (*p* < 0.01) AsA concentration in extracts of *C. reticulata* (7.72 ± 0.97 mg L^−1^) followed by *C. clementina* (4.37 ± 0.12 mg L^−1^) and *C. japonica* (1.39 ± 0.07 mg L^−1^) cultivars.

Finally, in the seeds of *C. reticulata*, the AsA amount was almost twice higher (*p* < 0.01) than that found in *C. japonica* (20.61 ± 0.57 mg L^−1^ and 12.78 ± 0.45 mg L^−1^, respectively).

### 3.5. Relationship among Bioactive Compounds in the Seeds

The correlations among bioactive compounds in seeds are reported in Table 1. *C. reticulata* showed a significant positive correlation between antioxidant capacity and total chlorophyll, antioxidant capacity and carotenoids as well as between total chlorophylls and carotenoids. *C. japonica* exhibited a significant negative correlation between antioxidant capacity and ascorbic acid and a significant positive correlation between chlorophylls and carotenoids.

## 4. Discussion

In this study, a comparison between different Citrus varieties was carried out to quantify the content of some bioactive compounds in the peel, pulp and seeds. Data collected allowed us to screen the most promising Citrus cultivar in terms of nutraceutical compounds and to valorise the parts of the fruit, such as peel and seeds, generally considered useless by-products of the production chain. Among cultivars, compelling experimental evidence emerges mainly from *C. reticulata* cultivar. The Phlegraean mandarin is a typical product of the volcanic Phlegraean area (Southern Italy, Naples), characterized by mild climate conditions and very fertile soils. This cultivar is not widespread outside the Campania region boundaries, obscured by the most famous Clementine and Kumquat varieties, with a broader national market.

From our data, it is evident that all fruit parts of the investigated citrus species showed strong antioxidant properties, especially peels and seeds, considered waste products, with limited or even without market value for the food industry [18,19].

Consistent with findings of other authors [34], the total pigment content was very high in the peel compared to the pulp in all tested varieties of mandarin, confirming the valuable antioxidant properties of this fruit part. Among cultivars, *C. clementina* showed the highest pigment content in both peel and pulp.

It is interesting to note that in seeds, *C. japonica* presented the highest content of carotenoids and chlorophylls compared to *C. reticulata*. This difference may be due to intrinsic characteristics of the species. It has been demonstrated that *Citrus* fruits are complex sources of pigments, especially carotenoids, with a broad diversity among the different species and cultivars in terms of types and amounts [35,36,37]. Therefore, most *Citrus* species show the same carotenoid profile, although some of them have higher concentrations [38].

Generally, the carotenoids are absent in seeds compared to peel and pulp, conversely to the concentration of chlorophylls. The presence of chlorophyll in the seeds of both *C. reticulata* and *C. japonica* cultivars is due to the chlorophyllous embryo. Especially in Kumquat, the cotyledon primordia were particularly evident under the outer seed integument (personal observation). The amount of chlorophyll in seeds is a valuable attribute because the chlorophyll presence ensures the activation of light capture mechanisms, as soon as germination begins [39]. Moreover, there are also some references about fruit photosynthesis: this process may be affected by light able to penetrate in fruit, temperature and ontogeny [40]. It is interesting to consider that the occurrence of a slight level of photosynthesis in mandarin fruit could determine the enrichment of the fruit biochemical profile.

Differently from chlorophylls, the presence of carotenoids in seeds and non-green tissues is common in many species such as maize, pumpkins, sunflowers [41].

The occurrence of carotenoids in plant tissues is associated with a protective function. They develop more abundantly in seedlings at a later time, to enlarge the sunlight harvesting and to defend the photosynthetic apparatus from damages due to excess of light. The carotenoids contribute, as antioxidants, to contrast the deterioration of the membranes induced by free radicals and ageing [42,43].

The function of carotenoids in the seed is less clear than in other plant tissues; however, previous researches have demonstrated that carotenoid presence in the grain is essential for the production of abscisic acid (ABA) and the induction of seed dormancy [44]. The dormancy is one of the mechanisms by which plants can delay germination when the environmental conditions are unfavourable to sprouting [45]. Generally, the number of plants with dormant seeds increases with increasing distance from the equator, in response to seasonality and habitat diversity [46]. It may be hypothesised that the higher content of carotenoids in the *C. japonica* seeds serves for higher production of ABA, in response to the plant’s need to maintain seeds quiescent for a longer time compared to *C. reticulata*. On the other hand, *C. reticulata* does not require a quiescent strategy for seeds because it is particularly diffused in Mediterranean ecosystems where the mild climate favours germination conditions.

Furthermore, carotenoids in seeds also contribute to the antioxidant defence of embryonal tissues limiting the membrane deterioration due to free radical and ROS production during seed ageing [42,43]. The presence of carotenoids ensures healthy and long-lived mandarin seeds and together with chlorophylls contributes to improve the fruit value, as chlorophyll and carotenoids are important antioxidant compounds in the human diet [47].

The most relevant result of our study regards the antioxidant power found in mandarin by-products. Our data demonstrate that considerable amounts of polyphenols, water-soluble antioxidants and ascorbic acid were found in the peel and extremely high concentrations of these compounds were measured in seeds, especially in *C. reticulata* cultivar. The peels, and even more so the seeds, are considered wastes of the citrus industry and currently to our knowledge, they are not used for the human diet, but only for ruminant nutrition [22].

The peel is generally the tissue richer in polyphenols and antioxidant activity than other fruit parts [26], even if the antioxidant properties are strictly related to the species. Our results evidence an interesting exception for the *C. reticulata* cultivar, where the highest concentration of total polyphenols and the most elevated antioxidant capacity was found in the seeds. According to other authors [48], we assume the elevated content of compounds with antioxidant action, located in particular in peel and seeds, represents a defence mechanism of the fruit and the embryo against external agents. Indeed, it is well known that these compounds are involved in the protection of the fruit and the embryo against herbivores and fungal pathogen attacks [49].

For the highest cumulative capacity to scavenge free radicals compared to peel and pulp, the seeds of *C. reticulata* are worthy of particular attention. The concurrent increase in total polyphenol and ascorbic acid contents suggests that these molecules may, in part, contribute to the seed antioxidant power in this species. The *C. japonica* seeds exhibited different nutraceutical traits compared to *C. reticulata*, showing a lower antioxidant capacity and polyphenol and ascorbic acid content, but a higher amount of chlorophylls and carotenoids. *C. reticulata* seeds also showed a positive relationship among chlorophylls, carotenoids and antioxidant capacity, while *C. japonica* revealed a positive relationship between chlorophylls and carotenoids but a negative relationship between ascorbic acid and antioxidant capacity, indicating that ascorbic acid does not contribute so much to the antioxidant power.

The absence of relationships among ascorbic acid and antioxidant capacity in the peel and pulp of mandarin cultivars suggests that other compounds should be responsible for the antioxidant power of these fruit components [27].

Several studies report that various phenolic compounds, compared to ascorbic acid, mainly influence antioxidant activity [50,51]. However, according to the literature [6], it may be argued that the phenolic compounds in citrus fruits contribute less than vitamin C to the antioxidant activity. This evidence seems to be confirmed for the seeds of the *C. reticulata* cultivar, where we have found a very high content of vitamin C. However, it cannot be excluded that other antioxidant compounds, such as flavonoids may be present in seeds enhancing the antioxidant power. In addition, other factors may contribute to this distinctive trait in Phlegrean mandarin, such as the plant age or the stage of fruit ripeness [52]. It is noteworthy that antioxidant properties found in the different parts of mandarin fruit are strictly related to intrinsic species characteristics. In fact, conversely to Phlegrean mandarin, in other citrus fruits such as orange, lemon and grapefruit, the higher amount of phenolic compounds, flavonoids, vitamin C, and antioxidant activity, were found in the peels compared to the inner wasted parts (pulp and seeds) [26]. Starting from this evidence, the high antioxidant charge of Phlegrean mandarin seeds assumes a great commercial value for diverse purposes. Lyophilised or fresh seeds extracts might be used as food additives or included in pharmaceutical formulations as promising alternatives to the common preparations [53]. The current challenge is to improve the extraction techniques of bioactive compounds from vegetable wastes to preserve the properties of these molecules over time and obtain a full by-product valorisation.

## 5. Conclusions

The comparison among diverse mandarin cultivars and fruit components revealed some important insights about the nutraceutical value associated with species and fruit parts. For all tested cultivars the peel is more abundant in antioxidants compared to pulp and may be potentially used as a dietary supplement. However, the most significant result concerns the seeds of the Phlegraean mandarin, which have proved to be highly rich in polyphenols, ascorbic acid, and antioxidant activity, compared to the other parts of the fruit and other citrus varieties.

Seeds could be inexpensive and readily available resources of bioactive compounds (such as natural antioxidants) for use in the food and pharmaceutical industries. The seed consumption would also reduce the problem of large amounts of wastes derived annually from the agri-food industry.

A sustainable re-utilisation of seeds and peels for industrial and pharmacological applications could represent a strong boost toward circular economy initiatives in Southern Italy.

Further analyses are needed to improve this initial research, allowing a deepen characterisation of bioactive molecules responsible for the high antioxidant power of the seeds.

From the economic point of view, the evidence that the Phlegraean mandarin is richer in bioactive compounds than the most commercialised varieties (i.e., kumquat and clementine) promotes the valorisation of a potentially unexploited resource typical of the Campania region (Southern Italy), whose economy is mainly based on the tourism and agriculture.

## Figures and Tables

**Table 1:** Pearson correlations among bioactive compounds in the seeds (* *p* < 0.05; ** *p* < 0.01).; total polyphenols—TP; antioxidant capacity—AC; total chlorophyll—Chl (a + b); total carotenoids—Car (x + c); ascorbic acid—AsA.

Cultivar	Variables	TP	AC	Chl (a + b)	Car (x + c)	AsA
*C. reticulata*	TP	1	0.373	0.094	0.096	−0.09
	AC		1	0.893 *	0.897 *	0. 265
	Chl (a + b)			1	0.998 **	0.544
	Car (x + c)				1	0.548
	AsA					1
*C. japonica*	TP	1	0.643	0.496	0.469	−0.414
	AC		1	−0.268	−0.239	−0.893 *
	Chl (a + b)			1	0.837 *	0.546
	Car (x + c)				1	0.314
	AsA					1
